# IAN Family Critically Regulates Survival and Development of T Lymphocytes

**DOI:** 10.1371/journal.pbio.0040103

**Published:** 2006-03-07

**Authors:** Takeshi Nitta, Mariam Nasreen, Takafumi Seike, Atsushi Goji, Izumi Ohigashi, Tadaaki Miyazaki, Tsutomu Ohta, Masamoto Kanno, Yousuke Takahama

**Affiliations:** **1**Division of Experimental Immunology, Institute for Genome Research, University of Tokushima, Tokushima, Japan; **2**Division of Molecular Immunology, Institute for Genetic Medicine, Hokkaido University, Sapporo, Japan; **3**Medical Genomics Center, National Cancer Center Research Institute, Chuo-ku, Tokyo, Japan; **4**Department of Immunology, Graduate School of Biomedical Sciences, Hiroshima University, Minami-ku, Hiroshima, Japan; National Jewish Medical and Research Center/Howard Hughes Medical InstituteUnited States of America

## Abstract

The IAN (immune-associated nucleotide-binding protein) family is a family of functionally uncharacterized GTP-binding proteins expressed in vertebrate immune cells and in plant cells during antibacterial responses. Here we show that all eight *IAN* family genes encoded in a single cluster of mouse genome are predominantly expressed in lymphocytes, and that the expression of *IAN1, IAN4,* and *IAN5* is significantly elevated upon thymic selection of T lymphocytes. Gain-of-function experiments show that the premature overexpression of *IAN1* kills immature thymocytes, whereas short hairpin RNA-mediated loss-of-function studies show that *IAN4* supports positive selection. The knockdown of *IAN5* perturbs the optimal generation of CD4/CD8 double-positive thymocytes and reduces the survival of mature T lymphocytes. We also show evidence suggesting that IAN4 and IAN5 are associated with anti-apoptotic proteins Bcl-2 and Bcl-xL, whereas IAN1 is associated with pro-apoptotic Bax. Thus, the IAN family is a novel family of T cell–receptor-responsive proteins that critically regulate thymic development and survival of T lymphocytes and that potentially exert regulatory functions through the association with Bcl-2 family proteins.

## Abstract 2

The authors describe new roles for IAN proteins (a previously uncharacterized family of GTP binding proteins) in T cell development; different IAN proteins play important roles at distinct stages of T cell development.

## Introduction

The development of T lymphocytes in the thymus involves a series of checkpoints, including T cell receptor (TCR)–mediated positive and negative selection. Positive selection ensures the selective survival of potentially useful T cells, whereas negative selection deletes harmful T cells, avoiding autoimmunity. Differential ligand–TCR interactions that result in positive and negative selection initiate differential intracellular signals that, in turn, lead to the survival-or-death decision of immature thymocytes [ 1– 3]. The Bcl-2 family proteins are known to play crucial roles in regulating the survival and apoptosis of developing thymocytes. Anti-apoptotic Bcl-2 family members, such as Bcl-2, Bcl-xL, and Mcl-1, support the survival and development of T lymphocytes [ 4– 6], whereas pro-apoptotic members, such as Bax, Bak, and Bim, essentially mediate the deletion of self-reactive thymocytes [ 7, 8]. However, how the TCR signals result in the regulation of thymocyte fate by the Bcl-2 family members is unclear.

To better understand the molecular mechanisms that regulate T-lymphocyte development and selection, an oligonucleotide microarray was screened for mouse cDNA that was highly expressed in positive-selector TCR-transgenic thymocytes. We found that *IAN1* and *IAN4* are expressed upon the positive selection of thymocytes. *IAN1* and *IAN4* belong to the recently discovered *IAN* (immune-associated nucleotide-binding protein)/ *GIMAP* (GTPase of the immunity-associated protein) family of genes that encode functionally unknown GTP-binding proteins expressed in immune tissues [ 9– 29]. It has been shown that *IAN1* expression is predominant in the lymphoid cells, increased upon thymocyte maturation [ 9, 10], and decreased in human T-leukemia cells [ 11], whereas the expression of mouse *IAN2*/ *imap38* is elevated in the spleens of mice infected with the experimental malarial parasite Plasmodium chabaudi [ 12– 14]. It has been also shown that *IAN4* and *IAN5* are highly expressed in leukemia cells in mouse and human, respectively [ 15, 22], whereas human *IAN5* inhibits the apoptosis induced by okadaic acid [ 21]. However, the function of the IAN family genes in lymphocyte development has been poorly characterized.

Thus far, the most conclusive results on the role of the IAN family members come from studies of the BB rat, an animal model for type I diabetes [ 16– 20, 25– 27]. The BB rat spontaneously develops insulin-dependent diabetes and exhibits lifelong T lymphopenia in which the numbers of peripheral CD4 ^+^ and CD8 ^+^ T cells are severely reduced [ 30, 31]. Recent studies have identified a frame-shift mutation in *IAN5,* which is responsible for the T lymphopenia in the BB rat [ 16– 20]. It has been further reported that *IAN5* is involved in the regulation of T cell activation [ 25] and in the post-thymic development of CD4 ^+^ CD25 ^+^ regulatory T cells [ 26]. These findings suggest that *IAN5* plays an important role in the maintenance and regulation of peripheral T lymphocytes.

The present study shows that the mouse genome encodes eight functional IAN genes within a tight cluster, and that among the eight IAN family members, *IAN1, IAN4,* and *IAN5* are highly expressed in T lymphocytes, and their expression is significantly elevated in immature thymocytes upon TCR-mediated positive selection. Retroviral overexpression and short hairpin RNA (shRNA)–mediated knockdown in developing thymocytes indicate that *IAN1, IAN4,* and *IAN5* critically and differentially influence the survival and development of T lymphocytes. The knockdown of *IAN4* and *IAN5* disturbs thymocyte development at different stages, whereas the premature overexpression of *IAN1* induces the apoptosis of immature thymocytes. It is further suggested that IAN4 and IAN5 are associated with anti-apoptotic proteins Bcl-2 and Bcl-xL, whereas IAN1 is associated with pro-apoptotic Bax. Importantly, Bcl-xL was found to interfere with the *IAN5*-mediated regulation of T cell survival. Taken together, these results indicate that the IAN family genes encode a novel family of TCR-responsive proteins that critically regulate the survival and death of developing T lymphocytes and that potentially do so via the association with Bcl-2 family proteins.

## Results

### Mouse IAN Gene Cluster Encodes Eight Proteins with GTP-Binding Motifs

**Figure 1:**
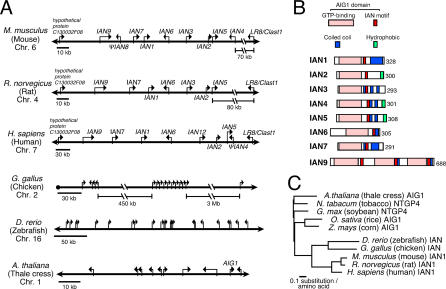
IAN Family Genes (A) The cluster of IAN family genes in the genome of indicated species. Mouse IAN genes and their orthologs in human and rat are indicated. For chicken, zebrafish, and thale cress, arrows indicate genes that putatively encode AIG1-domain-containing proteins. In chicken, 19 genes are predicted to encode AIG1 domain–containing proteins, and arrows indicate 15 genes clustered on Chromosome 2. In zebrafish, a cluster of 23 genes was found on Chromosome 16. In thale cress, ten out of 14 predicted genes are clustered on Chromosome 1. (B) Predicted structures of mouse IAN family proteins. Numbers refer to amino acid residues of full-length proteins. (C) A neighbor-joining tree of the AIG1 domain of IAN proteins. A. thaliana AIG1, residues 44–243; N. tabacum NTGP4 (AAD09518), residues 23–222; G. max NTGP4 (BI316235), residues 1–118; O. sativa AIG1 (CAE04223), residues 31–230; *Z. mays* AIG1 (AW120061), residues 1–200; D. rerio IAN (BC053197), residues 1–200; G. gallus IAN (XP_427942), residues 3–202; M. musculus IAN1, residues 31–230; R. norvegicus IAN1, residues 31–230; H. sapiens IAN1, residues 45–244. No IAN genes were found in the genomes of Drosophila melanogaster (fly), Anopheles gambiae (mosquito), Ciona intestinalis (sea squirt), Caenorhabditis elegans (nematode), Saccharomyces cerevisiae (yeast), and all bacteria and archea.

Using oligonucleotide microarray analysis, we screened for mouse cDNA that was highly expressed in positive-selector *H-2 ^b^* AND-TCR–transgenic thymocytes [ 32]. In addition to previously characterized genes such as *CCR7,* we found that *IAN1* and *IAN4* were expressed at higher levels in the positive-selector thymocytes than in the wild-type thymocytes ( Table S1). In the mouse genome, eleven IAN genes were previously predicted to lie within a tight cluster on Chromosome 6 [ 17]. By analyzing the nucleotide sequences of BAC clones and PCR-amplified C57BL/6 genomic DNA, we identified mouse *IAN1, IAN2, IAN3, IAN4, IAN5, IAN6,* and *IAN7* ( Figure 1A), which were transcribed in immune tissues ( Figure S1A). Provisional *IAN8* was identified to be a pseudogene, because it had stop codons and multiple frame shifts in possible coding frames, and its transcript was undetected ( Figure S1B). Provisional *IAN9, IAN10,* and *IAN11* were transcribed and spliced into a single mRNA encoding a protein with three GTP-binding motifs ( Figure 1B, Figure S2), and thus renamed *IAN9,* in agreement with the recently described human *gimap8* [ 28] and rat *IAN9* [ 29].

Figure 1B shows the predicted structures of all the eight members of the IAN family proteins in mice. The AIG1 domain [ 22] that contains a GTP-binding motif and a functionally undefined RxxxθNN[R,K][A,E] (θ, hydrophobic amino acids) sequence, designated as the IAN motif, was found in all the members. All members except IAN2 carried the coiled-coil motif. *IAN4* and *IAN5* showed markedly high similarity in amino acids (83.8% identity in 291 aa) and in ORF nucleotides (88.9% identity in 873 bp) ( Figure S3).

In addition to mouse, every vertebrate examined so far contained the IAN family gene cluster ( Figure 1A and 1C), whereas no IAN genes were found in the genomes of invertebrates and microorganisms that were registered in the public databases of NCBI and Ensembl, suggesting that the IAN family genes may play a role in the adaptive immune system in vertebrates. Interestingly, a cluster of the IAN family genes was found in the genome of Arabidopsis thaliana ( Figure 1A), and this cluster included *AIG1,* the expression of which was elevated upon infection by pathogenic bacteria [ 33]. Other higher plants also carried the IAN family genes ( Figure 1C).

### *IAN1, IAN4,* and *IAN5* Expression Is Increased upon Positive Selection of Thymocytes

**Figure 2:**
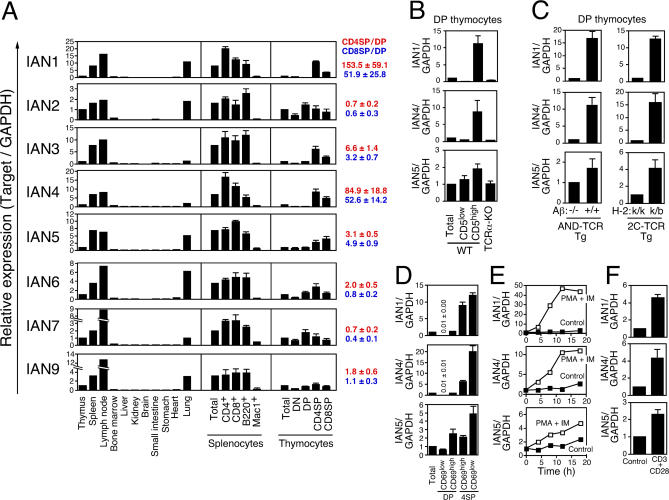
Expression of Mouse IAN Family Genes (A) Quantitative RT-PCR analysis of total RNA from C57BL/6 mouse tissues, purified splenocyte subsets, and purified thymocyte subsets. The mRNA levels of IAN family genes were initially normalized to *GAPDH* levels, and were further normalized to the levels expressed in the thymus. Relative expression of all IAN family genes in the thymus tissue is indicated as 1. (B) Relative mRNA levels of *IAN1, IAN4,* and *IAN5* in CD4 ^+^CD8 ^+^, CD4 ^+^CD8 ^+^CD5 ^low^, and CD4 ^+^CD8 ^+^CD5 ^high^ thymocytes from C57BL/6 (wild-type) mice and CD4 ^+^CD8 ^+^ thymocytes from TCRα-deficient mice [ 42]. (C) *IAN1, IAN4,* and *IAN5* mRNA levels in CD4 ^+^CD8 ^+^ thymocytes from positive selector (AND-TCR Aβ ^+/+^ and 2C-TCR ^k/b^ [ 43]) TCR-transgenic mice and null selector (AND-TCR Aβ ^−/−^ [ 44] and 2C-TCR ^k/k^) TCR-transgenic mice. (D) Relative mRNA levels of *IAN1, IAN4,* and *IAN5* in total, CD4 ^+^CD8 ^+^CD69 ^low^, CD4 ^+^CD8 ^+^CD69 ^high^, CD4 ^+^CD8 ^−^CD69 ^high^ and CD4 ^+^CD8 ^−^CD69 ^low^ thymocytes from C57BL/6 mice. (E) Thymocytes from TCRα-deficient mice were cultured with or without phorbol 12-myristate 13-acetate (0.2 ng/ml) and ionomycin (0.2 μg/ml) for the indicated periods. (F) Thymocytes from Aβ ^−/−^ β2m ^−/−^ mice [ 45] were cultured with or without plate-bound anti-CD3 (clone 2C11) and anti-CD28 (clone 37.51) antibodies for 24 h. Bar graphs show means ± standard errors.

Quantitative real-time PCR (polymerase chain reaction) revealed that all eight members of the *IAN* family in mouse were expressed abundantly in the spleen and the lymph node, followed by the thymus, bone marrow, and lung, but were poorly expressed in other tissues ( Figure 2A). In the spleen, all the IAN genes were predominantly expressed in CD4 ^+^, CD8 ^+^, or B220 ^+^ lymphocytes rather than in Mac1 ^+^ myeloid cells, and the expression of *IAN1, IAN4, IAN5,* and *IAN7* was higher in T lymphocytes than in B lymphocytes ( Figure 2A). In the thymus, the expression of *IAN1* and *IAN4* was robustly elevated during the development of CD4/CD8 double-positive (DP) thymocytes into CD4 single-positive (SP) and CD8SP thymocytes ( Figure 2A), in agreement with the initial microarray results ( Table S1). Among the other IAN family members, the expression of *IAN3* and *IAN5* was moderately but significantly increased during the development of DP cells into SP cells ( Figure 2A). Thus, *IAN1, IAN4,* and *IAN5* are highly expressed in T lymphocytes, and their expression is significantly elevated upon the maturation of DP to SP thymocytes.

The expression of *IAN1, IAN4,* and *IAN5* was higher in DP CD5 ^high^ thymocytes than in DP CD5 ^low^ thymocytes or TCRα-deficient DP thymocytes ( Figure 2B), and higher in positive-selector DP thymocytes than in null-selector DP thymocytes in TCR-transgenic mice ( Figure 2C). In normal mouse thymocyte subpopulations, the expression of *IAN1, IAN4,* and *IAN5* was increased in accordance with the positive selection of immature DP thymocytes to mature SP thymocytes, including the increase along the early events during the development of DP CD69 ^low^ cells to DP CD69 ^high^ cells and the increase along the late events during the development of CD4SP CD69 ^high^ cells to CD4SP CD69 ^low^ cells ( Figure 2D). In vitro stimulation of TCRα-deficient DP thymocytes with phorbol 12-myristate 13-acetate and ionomycin resulted in marked increases in *IAN1* and *IAN4* expression and a modest increase in *IAN5* expression ( Figure 2E). Similarly, in vitro stimulation of immature DP thymocytes, which were isolated from MHC class I and class II double-deficient mice, with anti-CD3 and anti-CD28 antibodies significantly increased *IAN1, IAN4,* and *IAN5* expression ( Figure 2F). Thus, the expression of *IAN1, IAN4,* and *IAN5* is increased during the positive selection of thymocytes.

### Premature Overexpression of *IAN1* Causes Apoptosis of DP Thymocytes

**Figure 3:**
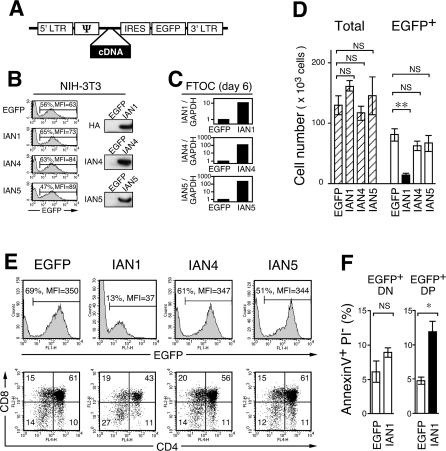
Overexpression of IAN1, IAN4, and IAN5 in Thymocyte Development (A) Diagram of MSCV retroviral constructs. IRES, internal ribosomal entry site; LTR, long terminal repeat; Ψ, packaging signal. (B) NIH-3T3 cells infected with retroviruses expressing IAN1-HA, IAN4, IAN5, or EGFP alone were analyzed for EGFP fluorescence by flow cytometry and for IAN protein expression by immunoblotting (IB). The frequency of cells and the mean fluorescence intensity (MFI) in the indicated area are shown. (C) Day 14.5 fetal thymocytes infected with indicated retroviruses were reconstituted in FTOC. EGFP ^+^ cells purified on day 6 were analyzed for mRNA expression. (D) Viable cell numbers of total cells (striped bars) and EGFP ^+^ cells (open bars) in FTOC on day 6. Filled bar indicates a significant reduction ( *p* < 0.01). EGFP, *n* = 7; IAN1, *n* = 6; IAN4, *n* = 6; IAN5, *n* = 6. (E) EGFP histograms of total cells and CD4/CD8 profiles of EGFP ^+^ cells in FTOC on day 6. Numbers in dot plots show the frequency of cells within boxes. (F) Annexin V and PI staining of indicated EGFP ^+^ thymocyte subpopulations in FTOC on day 6. Filled bar indicates a significant increase ( *p* < 0.05). Bar graphs show means ± standard errors. NS, not significant ( *p* ≥ 0.05); * *p* < 0.05; ** *p* <0.01. No significant difference in the CD4/CD8 developmental profiles was observed in EGFP ^−^ cell populations.

To study the roles of *IAN1, IAN4,* and *IAN5* in thymocyte development, CD4/CD8 double-negative (DN) immature thymocytes obtained from day 14.5 fetal mice were infected with retroviruses that overexpressed IAN genes along with enhanced green fluorescent protein (EGFP) ( Figure 3A– 3C) and examined for developmental capability in 2-deoxyguanosine-treated fetal thymus. It was found that the overexpression of *IAN1* reduced the number of EGFP ^+^ thymocytes ( Figure 3D) and the EGFP intensity of those EGFP ^+^ thymocytes ( Figure 3E). The increase in frequency of DN thymocytes and the decrease in frequency of DP thymocytes were modestly but significantly caused in the EGFP ^+^ cells by the *IAN1* overexpression ( Figure 3E). The frequency of apoptotic cells was significantly increased in *IAN1* overexpressing EGFP ^+^ DP thymocytes but not in EGFP ^+^ DN thymocytes ( Figure 3F). These effects of *IAN1* overexpression were detected only in EGFP ^+^ cells and not in coexisting EGFP ^−^ cells ( Figure 3D) that failed to express retrovirus-mediated genes and that comprised more than 30% of total thymocytes in the fetal thymus organ culture (FTOC) ( Figure 3E), indicating that the effects of *IAN1* overexpression were specific for the *IAN1*-overexpressing cells but not for co-existing EGFP ^−^ thymocytes, and that the *IAN1* overexpression affected thymocyte development in the presence of normally developing thymocytes. Thus, the premature overexpression of *IAN1* kills DP thymocytes, disturbing subsequent T cell development. By contrast, the overexpression of *IAN4* or *IAN5* did not significantly influence thymocyte development ( Figure 3D and 3E).

### Knockdown of *IAN4* and *IAN5* Differentially Disturbs Thymocyte Development

**Figure 4:**
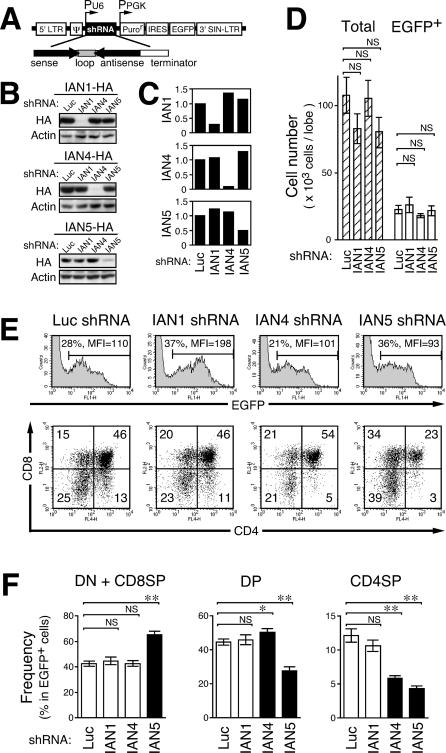
Knockdown of IAN1, IAN4, and IAN5 in Thymocyte Development (A) Diagram of retroviral shRNA constructs. Puro *^r^,* puromycin resistance gene. SIN-LTR, self-inactivating long terminal repeat. (B) BW5147 cells expressing IAN1-HA, IAN4-HA, or IAN5-HA were infected with shRNA retroviruses, and the infected cells were enriched by puromycin selection. Protein expression levels were analyzed by anti-HA IB. *Luciferase* (Luc) shRNA was used as control. (C) Day 14.5 fetal thymocytes infected with shRNA retroviruses were reconstituted in FTOC. EGFP ^+^ cells purified on day 6 were analyzed for mRNA expression. (D) Viable cell numbers of total cells (striped bars) and EGFP ^+^ cells (open bars) in FTOC on day 6. (E) EGFP histograms of total cells and CD4/CD8 profiles of EGFP ^+^ cells in FTOC on day 6. The frequency of EGFP ^+^ cells and the mean fluorescence intensity (MFI) in the indicated area are shown in the histograms. Numbers in dot plots show the frequency of EGFP ^+^ cells within boxes. (F) Frequencies of indicated cell populations on day 6. Filled bars indicate significant difference from the values in the control group ( *Luc* shRNA) ( *p* < 0.05). Bar graphs show means ± standard errors. NS, not significant ( *p* ≥ 0.05); ^*^ *p* < 0.05; ^**^ *p* < 0.01. In (D) and (F), *Luc* shRNA, *n* = 11; *IAN1* shRNA, *n* = 5; *IAN4* shRNA, *n* = 11; *IAN5* shRNA, *n* = 8. No significant difference in the CD4/CD8 developmental profiles was observed in EGFP ^−^ cell populations.

Loss-of-function examination of thymocyte development was carried out using the retrovirus-mediated RNA interference technique. In this technique, shRNA was expressed under the control of the PolIII-dependent U6 promoter ( Figure 4A). The shRNA markedly and specifically decreased the expression of *IAN1, IAN4,* and *IAN5* in immature T cells at both mRNA and protein levels ( Figure 4B and 4C). By contrast, the other IAN family members, *IAN2, IAN3, IAN6, IAN7,* and *IAN9,* were not affected by any of the shRNAs ( Figure S4), further indicating the specificity of the shRNA-mediated knockdown.

**Figure 5:**
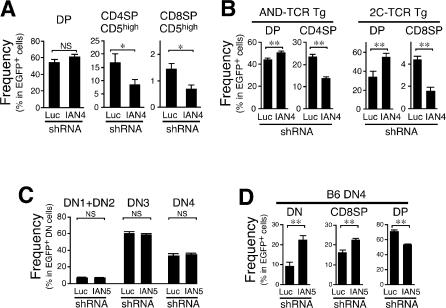
Differential Roles of IAN4 and IAN5 in Thymocyte Development (A) Day 14.5 fetal thymocytes infected with shRNA retroviruses were reconstituted in FTOC. Frequencies of indicated cell populations on day 10 are shown. *Luc* shRNA, *n* = 4; *IAN4* shRNA, *n* = 5. (B) Day 14.5 fetal thymocytes from AND-TCR-transgenic or 2C-TCR-transgenic mice were infected and reconstituted in C57BL/6 fetal thymus lobes for 6–8 d. For 2C-TCR–transgenic thymocytes, mature CD8SP cells were analyzed by gating CD8SP CD5 ^high^ population. AND- *Luc* shRNA, *n* = 11; AND- *IAN4* shRNA, *n* = 12; 2C- *Luc* shRNA, *n* = 5; 2C- *IAN4* shRNA, *n* = 5. (C) Day 14.5 fetal thymocytes infected with shRNA retroviruses were reconstituted in FTOC (as shown in Figure 4C– 4F). EGFP ^+^ thymocytes on day 6 were analyzed for DN subpopulations by staining with anti-CD25 and anti-CD44 antibodies. *Luc* shRNA, *n* = 6; *IAN5* shRNA, *n* = 8. (D) CD4 ^−^CD8 ^−^CD25 ^−^CD44 ^−^ DN4 cells were purified from day 17 fetal thymocytes by depleting the cells expressing CD4, CD8, CD25, or CD44, infected with indicated retroviruses, and reconstituted in FTOC for 2 d; *n* = 4. Bar graphs show means ± standard errors. NS, not significant ( *p* ≥ 0.05); ^*^ *p* < 0.05; ^**^ *p* < 0.01.

DN immature thymocytes were infected with retroviruses that expressed these shRNA along with EGFP, and were transferred into 2-deoxyguanosine-treated fetal thymus. The infection with the shRNA retroviruses did not affect cell culture conditions or the development of uninfected cells, as no significant effects were detected on the number of developing thymocytes in the EGFP ^+^ fractions as well as in the EGFP ^−^ fractions ( Figure 4D) or on the developmental profiles of EGFP ^−^ thymocytes (unpublished data). It was found that *IAN1* shRNA had no significant effects on the CD4/CD8 profile of EGFP ^+^ cells, whereas *IAN4* shRNA caused a significant decrease in the generation of EGFP ^+^ CD4SP thymocytes, which paralleled the increase in frequency of EGFP ^+^ DP cells ( Figure 4E and 4F). In agreement with the thymocyte frequency, the cellularity of the EGFP ^+^ CD4SP subpopulation was significantly reduced by the *IAN4* shRNA expression (2.4 × 10 ^3^ ± 0.3 × 10 ^3^ by the control *luciferase* shRNA expression, and 1.0 × 10 ^3^ ± 0.2 × 10 ^3^ by the *IAN4* shRNA expression; *p* < 0.001). By contrast, the cell number of the EGFP ^+^ DP subpopulation was not significantly affected by the *IAN4* shRNA expression (9.4 × 10 ^3^ ± 1.3 × 10 ^3^ by the control *luciferase* shRNA expression, and 8.3 × 10 ^3^ ± 1.3 × 10 ^3^ by the *IAN4* shRNA expression; not significant). Furthermore, *IAN4* shRNA significantly reduced the generation of CD8SP CD5 ^high^ and CD4SP CD5 ^high^ mature T cells but not that of DP thymocytes within the EGFP ^+^ population ( Figure 5A). The introduction of *IAN4* shRNA significantly decreased the generation of AND-TCR–transgenic CD4SP T cells and 2C-TCR–transgenic CD8SP CD5 ^high^ T cells ( Figure 5B). Thus, *IAN4* is required for supporting the positive selection of CD4 T cells and CD8 T cells.

On the other hand, the introduction of *IAN5* shRNA most markedly decreased the frequency of DP and CD4SP cells and increased that of DN and CD8SP immature cells within the EGFP ^+^ population ( Figure 4E and 4F). In agreement with the frequency, the cellularity of EGFP ^+^ DP and EGFP ^+^ CD4SP subpopulations was significantly reduced by the *IAN5* shRNA expression (EGFP ^+^ DP cells: 9.4 × 10 ^3^ ± 1.3 × 10 ^3^ by the control *luciferase* shRNA expression, and 6.0 × 10 ^3^ ± 0.9 × 10 ^3^ by the *IAN5* shRNA expression ( *p* < 0.05); EGFP ^+^ CD4SP cells: 2.4 × 10 ^3^ ± 0.3 × 10 ^3^ by the control *luciferase* shRNA expression, and 0.9 × 10 ^3^ ± 0.2 × 10 ^3^ by the *IAN5* shRNA expression ( *p* < 0.001)). By contrast, the cell number of EGFP ^+^ DN and EGFP ^+^ CD8SP immature subpopulations was elevated by the *IAN5* shRNA expression (8.6 × 10 ^3^ ± 1.0 × 10 ^3^ by the control *luciferase* shRNA expression, and 12.4 × 10 ^3^ ± 1.4 × 10 ^3^ by the *IAN5* shRNA expression; *p* < 0.05). The distribution of DN subpopulations defined by CD25 and CD44 in the EGFP ^+^ cells was not affected by the infection of day 14.5 fetal thymocytes that were mostly DN1 and DN2 before the culture, with the *IAN5* shRNA retrovirus ( Figure 5C), whereas the infection of purified DN4 thymocytes with the *IAN5* shRNA retrovirus significantly reduced the generation of EGFP ^+^ DP cells but significantly increased the numbers of EGFP ^+^ DN and EGFP ^+^ CD8SP immature cells in 2-d FTOC ( Figure 5D). These results indicate that *IAN5* is required for the optimal generation of DP thymocytes.

### Association of *IAN1, IAN4,* and *IAN5* with Bcl-2 Family Proteins

**Figure 6:**
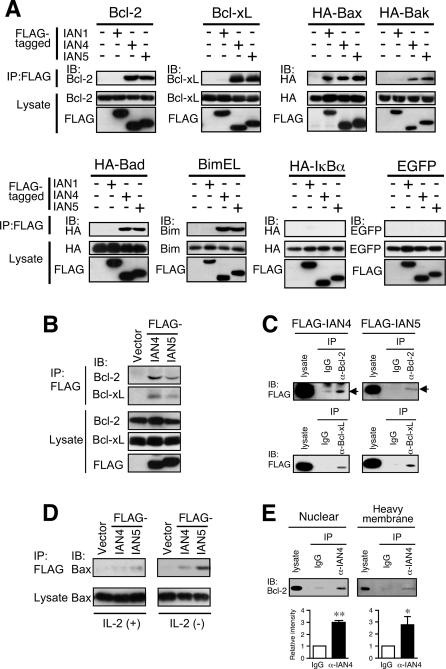
Interaction of IAN Family Proteins with Bcl-2 Family Proteins (A) 293T cells were co-transfected with FLAG-tagged IAN molecules together with Bcl-2, Bcl-xL, HA-tagged Bax, HA-tagged Bak, HA-tagged Bad, BimEL, HA-tagged IκBα, or EGFP. Cell lysates were IP with anti-FLAG M2 antibody and IB with indicated antibodies. (B) 23–1–8 T cells expressing EGFP alone (Vector), FLAG-tagged IAN4, or FLAG-tagged IAN5 were IP with anti-FLAG M2 antibody and IB with anti-Bcl-2 or anti-Bcl-xL antibody. (C) 23–1–8 T cells expressing FLAG-tagged IAN4 or FLAG-tagged IAN5 were IP with normal IgG or anti-Bcl-2 or anti-Bcl-xL antibody and IB with anti-FLAG M2 antibody. Arrows indicate FLAG-tagged IAN4 or FLAG-tagged IAN5. (D) 23–1–8 T cells expressing EGFP alone (Vector), FLAG-tagged IAN4, or FLAG-tagged IAN5 were cultured in the presence or absence of IL-2 for 36 h. Cell lysates were IP with anti-FLAG M2 antibody and IB with anti-Bax antibody. (E) Nuclear and heavy membrane fractions prepared from 23–1–8 T cells were lysed in buffer containing 1% CHAPS. The lysates were IP with normal rabbit IgG or anti-IAN4 antibody and IB with anti-Bcl-2 antibody. Means and standard errors ( *n* = 4) of relative intensities of the bands were analyzed by using NIH Image software. ^*^ *p* < 0.05; ^**^ *p* < 0.01.

To gain an insight into the mechanisms underlying the regulation of thymocyte development by the IAN family members, an antibody array of signal transduction molecules was screened for proteins that could bind to IAN4, revealing the selective binding of the Bcl-2 family proteins, including anti-apoptotic Bcl-2 and Bcl-xL ( Figure S5). Subsequent examination with co-immunoprecipitation (IP) analysis using transfected 293T cells confirmed that either *IAN4* or *IAN5* interacted with Bcl-2 and Bcl-xL ( Figure 6A). In addition, IAN4 or *IAN5* also interacted with the pro-apoptotic Bcl-2 family members Bax, Bak, Bad, and BimEL in the transfected 293T cells. By contrast, *IAN1* was selectively associated with the pro-apoptotic member, Bax, and not with the other Bcl-2 family members tested ( Figure 6A). Two other unrelated proteins, IκBα and EGFP, did not interact with any of the IAN family proteins ( Figure 6A), highlighting the specificity of the interaction between the IAN family proteins and the Bcl-2 family proteins.

Within the BW5147 thymocyte lines that were transduced with the IAN family genes, *IAN4* and *IAN5* were predominantly localized in the intracellular membrane fractions, including the mitochondria and the endoplasmic reticulum ( Figure S6), in agreement with the localization of Bcl-2 and Bcl-xL ([ 34]; also shown in Figure S6B). On the other hand, IAN1 was found in the cytoplasmic fraction ( Figure S6), in agreement with the localization of Bax [ 34].

To study the interaction of the IAN family proteins with the endogenous Bcl-2 family proteins in T-lymphoid cells, we initially tested 23–1–8 T lymphocyte clones expressing FLAG-tagged *IAN4* or FLAG-tagged IAN5. IP of FLAG-tagged *IAN4* or *IAN5* with anti-FLAG antibody resulted in the co-precipitation of endogenous Bcl-2 or Bcl-xL ( Figure 6B). Reciprocally, endogenous Bcl-2 or Bcl-xL IP with anti-Bcl-2 or anti-Bcl-xL antibodies was co-precipitated with FLAG-tagged *IAN4* or *IAN5* ( Figure 6C). The pro-apoptotic member Bax was also co-precipitated with FLAG-tagged *IAN4* or *IAN5* in 23–1–8 T cells ( Figure 6D). This co-precipitation of Bax was more pronounced in the cells that initiated apoptosis upon IL-2 withdrawal, than in the cells exposed to IL-2 ( Figure 6D), in agreement with the apoptosis-associated translocation of Bax from the cytoplasm to the intracellular membrane fractions [ 34, 35] where *IAN4* and *IAN5* are predominantly localized ( Figure S6).

We then analyzed the interaction between the endogenous IAN family proteins and the endogenous Bcl-2 family proteins in 23–1–8 T lymphocytes. As shown in Figure 6E, IP of endogenous IAN4 with anti-IAN4 antibody led to the co-precipitation of a small fraction of endogenously expressed Bcl-2 in the intracellular membrane fractions such as the nuclear and heavy membrane fractions. These results indicate that the IAN family proteins are associated with the endogenous Bcl-2 family proteins in T lymphocytes.

### *IAN5* Regulates T Cell Survival and Apoptosis

**Figure 7:**
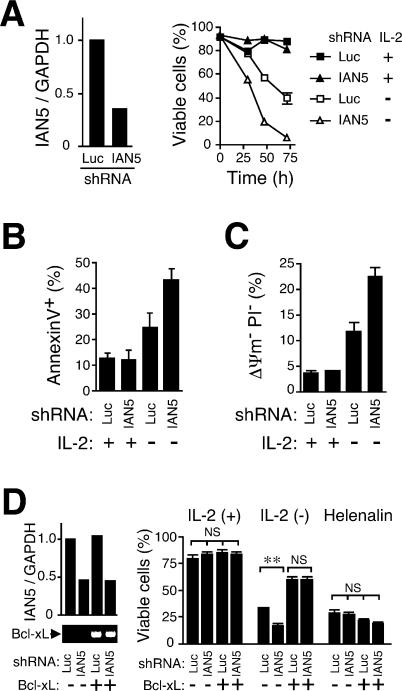
Knockdown of IAN5 in 23–1–8 T Lymphocytes (A) 23–1–8 T lymphocyte clones expressing shRNAs were analyzed for *IAN5* mRNA expression and cultured in the presence or absence of IL-2. Cell viability was quantified by PI staining and flow cytometry analysis. (B and C) Cells in 48-h culture were analyzed for apoptosis induction. Frequencies of Annexin-V-positive cells (B) or mitochondrial membrane potential (Δψm)-negative cells (C) are shown. (D) 23–1–8 T cells expressing shRNAs with or without human *Bcl-xL* were analyzed for *IAN5* expression by quantitative RT-PCR and for human *Bcl-xL* expression by conventional RT-PCR (left panel). Cells were cultured in the presence or absence of IL-2 for 72 h or in the presence of IL-2 and 5 μM helenalin for 48 h, and cell viability was quantified by PI staining (right panel). Graphs show means ± standard errors. NS, not significant ( *p* ≥ 0.05); ^**^ *p* < 0.01.

Based on the findings that both IAN4 and IAN5 are involved in the generation of T lymphocytes ( Figures 4 and 5) and are associated with the Bcl-2 family proteins ( Figure 6), we finally studied the role of these molecules in the survival and apoptosis of T lymphocytes. We found that the knockdown of *IAN5* in the IL-2-dependent 23–1–8 T cell line reduced the viability of the cells upon IL-2 withdrawal ( Figure 7A), whereas the IAN4 knockdown had no effect in this condition (unpublished data). Apoptosis as determined by Annexin V staining or the loss of mitochondrial membrane potential was also enhanced by the *IAN5* shRNA ( Figure 7B and 7C). These results indicate that *IAN5* is required for supporting the survival of T lymphocytes upon cytokine withdrawal.

The reduced survival of the IL-2–withdrawn 23–1–8 T cells by IAN5 shRNA was markedly restored by the additional expression of *Bcl-xL* ( Figure 7D). The overexpression of *Bcl-xL* failed to inhibit the apoptosis of 23–1–8 T cells induced by helenalin ( Figure 7D), an inducer of caspase-dependent apoptosis that occurs independent of Bcl-2-family–mediated mitochondrial signals [ 36], indicating that the Bcl-xL–mediated restoration of cell survival is specific for mitochondrial apoptosis [ 34]. These results indicate that the *Bcl-xL* overexpression specifically interferes with the enhanced apoptosis by IAN5 knockdown, suggesting the role of Bcl-2 family proteins in the IAN5-mediated regulation of T cell survival.

## Discussion

The present study shows that *IAN1, IAN4,* and *IAN5* are highly expressed in mature T lymphocytes and their expression is significantly elevated in DP thymocytes upon TCR-mediated positive selection. The shRNA-mediated knockdown of *IAN4* disturbed T cell development at the process of positive selection, whereas the knockdown of *IAN5* affected the earlier stage of T cell development at the generation of DP thymocytes. By contrast, the knockdown of *IAN1* did not significantly affect T cell development. On the other hand, the premature overexpression of *IAN1* induced the apoptosis of DP thymocytes, whereas the overexpression of *IAN4* or *IAN5* showed no significant effects on T cell development. These results indicate that *IAN1, IAN4,* and *IAN5* critically and differentially regulate the development of T lymphocytes in the thymus.

Our results suggest that IAN4 and IAN5 are associated with the Bcl-2 family proteins including anti-apoptotic Bcl-2 and Bcl-xL as well as pro-apoptotic Bax, whereas IAN1 is selectively associated with Bax. The overexpression of Bcl-xL restored the reduced survival of T cells caused by IAN5 deficiency, suggesting the role of Bcl-xL in the IAN5-mediated regulation of T cell survival. Our results also show that TCR signals in immature thymocytes upregulate the expression of *IAN1, IAN4,* and *IAN5*. Although it is possible that the expression and function of these IAN family members in developing thymocytes may also be regulated by the survival signals derived from other receptors, such as the IL-7 receptor and Notch-1, our results suggest that IAN1, IAN4, and IAN5 may relay TCR signals for apoptosis regulation by the Bcl-2 family members, critically controlling the survival and death of immature thymocytes and possibly regulating the repertoire selection of developing T cells in the thymus.

IAN4 and IAN5 are highly similar in terms of primary structure and subcellular localization. However, the knockdown of *IAN4* and *IAN5* differentially affected thymocyte development; i.e., IAN4 deficiency disturbed positive-selection–mediated SP thymocyte generation, whereas IAN5 deficiency affected DP thymocyte generation ( Figures 4 and 5). As the expression of *IAN5* but not *IAN4* was detected in the newly generated DP thymocytes before the TCR engagement (e.g., TCRα-deficient DP thymocytes in Figure 2B, and DP CD69 ^low^ thymocytes in Figure 2D), the differential effects of IAN4 deficiency and IAN5 deficiency may be due, at least in part, to their differential expression profiles in the developing thymocytes. On the other hand, it was also shown that the knockdown of *IAN5,* but not *IAN4,* enhanced the apoptosis of the IL-2–dependent T cell line upon IL-2 withdrawal, suggesting that IAN4 and IAN5 differentially regulate the survival of mature T cells that express both IAN4 and IAN5. The differential functions of IAN4 and IAN5 may also be applied to developing thymocytes. We are currently producing mice deficient in either IAN4 or IAN5 in order to elucidate the differential roles of IAN4 and IAN5 in thymocyte development and selection as well as mature T cell survival in vivo.

It was previously reported that IAN4 and IAN5 are highly expressed in leukemia cells [ 15, 22]. However, our results showed that the overexpression of either IAN4 or IAN5 did not significantly influence the cellularity or development of immature thymocytes in FTOC ( Figure 3). It is thus possible that in promoting cellular growth and/or oncogenesis, IAN4 and IAN5 may differentially affect immature thymocytes and other lymphoid cells including mature T lymphocytes. In vivo analysis of the long-term effects, including malignancy, of IAN4 or IAN5 overexpression in hematopoietic cells is in progress.

It was previously shown that a mutational loss of IAN5 in rat ( *lyp* mutation) causes T lymphopenia [ 16– 20], which leads to the development of insulin-dependent type I diabetes [ 30]. IAN5 deficiency in rat T lymphocytes causes mitochondrial dysfunction and spontaneous apoptosis [ 18]. However, the molecular mechanism underlying the regulation of T cell survival by IAN5 was not clarified. Our results indicate that IAN5 knockdown enhances the apoptosis of T lymphocytes upon cytokine withdrawal, and that the reduced survival of T cells by the IAN5 knockdown is restored by the overexpression of Bcl-xL. Our results also suggest that IAN5 is associated with the Bcl-2 family proteins. Together, the present results suggest that IAN5 expressed in T lymphocytes regulates the mitochondria-mediated apoptosis pathway through the interaction with the Bcl-2 family proteins.

Our results also show that the IAN5 knockdown perturbs the generation of DP thymocytes. This perturbation appears to occur during the differentiation of CD8SP immediate precursor cells to DP thymocytes ( Figure 5C and 5D). A similar phenotype of the thymocytes was described in vivo in IAN5-deficient rat, in which the number of DP thymocytes was reduced and that of DN thymocytes was increased [ 19]. Interestingly, this phenotype was found in not only *lyp*/ *lyp* rats but also *lyp*/+ rats [ 19], indicating that the haploinsufficiency of IAN5 affects thymocyte development. Indeed, our results show that the reduction of IAN5 mRNA level to approximately 50% could affect thymocyte development ( Figure 4C and 4F), supporting the notion that the amount of IAN5 expression critically regulates the generation of DP thymocytes.

The molecular basis for the association of the IAN family proteins with the Bcl-2 family proteins is still unclear. Our results show that either IAN4 or IAN5 interacts with endogenous Bax in apoptotic T cells rather than in healthy growing T cells even though the total amount of intracellular Bax expression is equivalent in apoptotic cells and growing cells ( Figure 6D). It was previously shown that Bax, which is localized in the cytosol of normal cells, responds to apoptosis-inducing stimuli by translocating onto the mitochondrial membrane where apoptotic reactions including ΔΨm loss and cytochrome-c release are operated [ 34, 35]. Thus, the interaction between Bax and IAN4 or IAN5 is likely regulated by the apoptotic stress-induced translocation of Bax to the mitochondria where IAN4 and IAN5 are localized. These results suggest that the association between the IAN family proteins and the Bcl-2 family proteins, including the pro-apoptotic proteins such as Bax, likely reflects a specific association with physiological relevance rather than merely a detection of nonspecific binding between overexpressed molecules. Accordingly, we detected the association between endogenous IAN4 and endogenous Bcl-2 in T cells. The specific association at the mitochondria between anti-apoptotic Bcl-xL and either IAN4 or IAN5 is further supported by our preliminary results that the deletion of the C-terminal hydrophobic region from either one of Bcl-xL, IAN4, or IAN5 caused the failure in their mitochondrial localization and in the association between Bcl-xL and either IAN4 or IAN5 (unpublished data). We are currently studying the structural basis for the intracellular association between the IAN family proteins and the Bcl-2 family proteins in detail to elucidate the molecular mechanisms of IAN-family–mediated apoptosis regulation.

Finally, our results showed that the premature overexpression of *IAN1* kills DP thymocytes. Our results also showed that unlike IAN4 and IAN5, IAN1 is selectively associated with pro-apoptotic Bax rather than anti-apoptotic Bcl-2 or Bcl-xL. Thus, IAN1 may be involved in regulating the apoptosis of immature DP thymocytes, possibly during the negative selection of self-reactive thymocytes. However, in agreement with previous reports [ 9, 10], our results showed that the expression of *IAN1* is increased during the positive selection of thymocytes. It is possible that differential TCR signals that determine the positive and negative selection of immature thymocytes may critically regulate the timing and amount of IAN1 expression; high-avidity and/or strong TCR engagement may result in a rapid and/or marked elevation of IAN1, which may cause the apoptosis of immature thymocytes, whereas low-avidity and/or weak TCR engagement may slowly and/or modest increase IAN1 expression, which may not be sufficient to kill positively selected thymocytes. We are currently studying the role of IAN1 in positive and negative selection in greater detail by using TCR-transgenic thymocytes.

In conclusion, the present results indicate that the IAN family genes encode a novel family of TCR-responsive proteins that critically regulate the survival and death of developing T lymphocytes. As the first described gene cluster conserved in vertebrates and higher plants, the IAN family may also provide clues to yet unknown self-defense mechanisms common in higher organisms.

## Materials and Methods

### Oligonucleotide microarray

Total RNA extracted from thymocytes of *H-2 ^b^* AND-TCR–transgenic mice and C57BL/6 mice was reverse-transcribed, in vitro transcribed in the presence of biotinylated UTP and CTP (Enzo Diagnostics, New York, New York, United States), and hybridized to an oligonucleotide array (Murine Genome U74Av2, U74Bv2, and U74Cv2, Affymetrix, Santa Clara, California, United States). Fluorescence intensities were captured with a laser confocal scanner (Hewlett-Packard, Palo Alto, California, United States), and analyzed with Microarray Suite Version 4.0 software (Affymetrix). Two independent experiments were performed to ascertain the reproducibility. Array data has been deposited in the Gene Expression Omnibus (GEO) database (accession number GSE3909).

### Bioinformatic analysis

Genome sequences were obtained from Celera database ( http://www.celera.com, NCBI public database ( http://www.ncbi.nih.gov, Ensembl database ( http://www.ensembl.org/index.html), and InterPro database ( http://www.ebi.ac.uk/interpro). AIG1 domain and coiled-coil motifs were identified using CD-Search ( http://www.ncbi.nlm.nih.gov/Structure/cdd/wrpsb.cgi, SMART ( http://smart.embl-heidelberg.de, and COILS ( http://www.ch.embnet.org/software/COILS_form.html). The neighbor-joining tree was drawn using GENETYX software (GENETYX, Tokyo, Japan).

### Retrovirus infection and FTOC

The retrovirus vector pMRX-IRES-EGFP [ 37] was used for the overexpression of cDNA in BW5147 cells or 23–1–8 cells. For the overexpression in fetal thymocytes, pMSCV-IRES-EGFP [ 38] was used. To construct retrovirus vectors expressing shRNA, oligonucleotides encoding stem-loop shRNA sequences ( Table S2) were inserted into the *Bam*HI- *Eco*RI site of pSIREN-RetroQ, which was purchased from BD Clontech, Mountain View, California, United States. For the expression of shRNA in fetal thymocytes, a DNA fragment encoding IRES-EGFP was obtained from pMRX-IRES-EGFP and inserted into the *Xho*I site of pSIREN-RetroQ to monitor the shRNA-expressing cells. Further construction details are available upon request.

Plat-E cells were transfected for retrovirus production [ 39]. BW5147, NIH-3T3, or 23–1–8 cells were infected with retroviruses in the presence of 10 μg/ml polybrene. Retrovirus infection of day 14.5 B6 fetal thymocytes was previously described [ 40]. DN4 thymocytes were purified from day 16.5 fetal thymocytes by depleting CD4, CD8, CD44, and CD25 using biotinylated antibodies and streptavidin-conjugated magnetic beads (Miltenyi Biotech, Bergisch Gladbach, Germany) (>97% purity). Retrovirus-infected fetal thymocytes were transferred into 2-deoxyguanosine–treated day 15.5 B6 fetal thymus lobes by hanging drop culture, and the reconstituted thymus lobes were cultured in conventional FTOC conditions [ 40].

### Flow cytometry analysis and cell sorting

Multicolor flow cytometry analysis and cell sorting were performed using FACS-Calibur or FACS-Vantage (BD Biosciences, San Jose, California, United States) as described [ 40, 41]. Cells with >95% purity were used. For the detection of apoptosis in developing thymocytes, cells were stained with phycoerythrin-conjugated Annexin V (MBL, Nagoya, Japan) before staining for CD4 and CD8. Mitochondrial membrane potential was analyzed by staining cells with 3,3′-dihexyloxacarbocynine iodide and propidium iodide (PI).

### Quantitative RT-PCR

Real-time RT-PCR was performed with the iQ SYBR Green Supermix and iCycler iQ Real Time PCR System (Bio-Rad, Hercules, California, United States). Amplified signals were confirmed to be single bands over gel electrophoresis, and normalized to GAPDH levels. Primer sequences are available upon request.

### Antibody production

Rabbits were immunized with synthetic peptides of mouse IAN4 (METLQNVVTGGKKGGC) and IAN5 (LQKSTYGTIVQGPEAHC) conjugated to KLH (MBL).

### Antibody array and IP analysis

Antibody array analysis was performed using Signal Transduction Array (Hypromatrix, Worcester, Massachusetts, United States) according to the manufacturer's instructions. For IP of FLAG-tagged proteins, cell lysates in 10 mM HEPES (pH 7.5), 150 mM NaCl, 1% CHAPS, and protease inhibitors were incubated with agarose-bead-conjugated anti-FLAG M2 antibody (Sigma, St. Louis, Missouri, United States). For IP with anti-Bcl-2 (BD Biosciences) or anti-Bcl-xL (MBL) antibodies, cell lysates were incubated with primary antibodies and agarose-bead-conjugated protein A or G. The beads were then washed and boiled in Laemmli gel-loading buffer before performing SDS-PAGE and IB. For IP with anti–Bcl-2 antibody, the cell lysis buffer contained 1% Triton X-100 instead of 1% CHAPS. Subcellular fractionation was performed as described [ 15].

## Supporting Information

Figure S1 *ΨIAN8* Is a Pseudogene
(A) Genomic localization of mouse *IAN* family genes spanning between previously predicted *IAN8* and *IAN4* is shown [ 17]. cDNAs prepared from thymocytes (Thy) and splenocytes (Spl) of C57BL/6 mice were PCR-amplified for indicated genes. Unlike the expression of the other *IAN* family genes, the expression of putative *IAN8* was not detected in thymocytes or splenocytes. A portion of this putative gene (360 bp) was also PCR-amplified from C57BL/6 genomic DNA (Gen) and sequenced.
(B) Nucleotide sequence and deduced three frames of amino acid sequences show multiple frame shifts, including a stop codon (nt 270–272, boxed) within possible coding frames (underlined). Provisional GTP-binding motifs (G1, G2, and G3) are boxed. Thus, this gene previously predicted as *IAN8* is a pseudogene. We renamed this locus *ΨIAN8*.
(446 KB PDF).

Figure S2 Mouse *IAN9* Gene
(A) Diagram of the genomic region between the hypothetical protein C130032F08 gene and *ΨIAN8*. A previous report predicted three genes *(IAN9, IAN10,* and *IAN11)* in this region [ 17], and we indeed found three putative ORFs encoding GTP-binding IAN proteins (GTP-binding motifs; open boxes). However, a computer search for these putative ORFs in the NCBI public database revealed a hypothetical transcript (accession number XM_144696) that covers the sequence ranging from the 5′-end of *IAN11* to the 3′-end of *IAN9* and encodes a protein with three GTP-binding motifs. Filled boxes represent its exons, with putative positions of start (ATG) and stop codons.
(B) To examine whether *IAN9, IAN10,* and *IAN11* comprise three independent genes or a single gene, we performed RT-PCR analysis with primers matching the 5′-end of *IAN11* and the 3′-end of *IAN9*. A 2.2-kb PCR product was detected from thymocytes (Thy) and splenocytes (Spl), and its nucleotide sequence was identical to that of the hypothetical transcript.
(C and D) Either probe 1 that matches *IAN11* or probe 2 that matches *IAN9* hybridized to a transcript of approximately 3 kb without any other transcripts in the Northern blot analysis. Thus, this genomic region in which the three IAN genes were previously predicted transcribes a single mRNA that encodes a putative 688 aa protein with three GTP-binding motifs. We renamed this gene *IAN9* (accession number AB178029).
(240 KB PDF).

Figure S3 Structural Similarity between IAN4 and IAN5 (A) Similarity in amino acid sequences among mouse IAN family proteins is summarized. Amino acid sequence homology was calculated with GENETYX software. IAN4 and IAN5 showed the highest similarity (as enclosed in a box).
(B) Sequence alignment of mouse IAN4 and IAN5.
Asterisks represent identical amino acids.
GTP-binding motifs (G1, G2, G3, and G4), IAN motifs, coiled-coil motifs, and hydrophobic regions are boxed.
(C) Membrane blots containing 3 μg per lane of poly(A) ^+^ RNA from the thymus and spleen of C57BL/6 mice were hybridized with ^32^P-labeled IAN4-ORF, IAN4-specific, IAN5-specific, or GAPDH probe. Total RNA was electrophoresed in the same gel, and the positions of 28S and 18S ribosomal RNAs are indicated.
Arrows indicate position and predicted size of the transcripts.
The two bands observed with the IAN4-ORF probe correspond to IAN4 and IAN5 transcripts.
(54 KB PDF).

Figure S4 Specific Knockdown of IAN1, IAN4, and IAN5 in Thymocyte Development Day 14.5 fetal thymocytes infected with shRNA retroviruses were reconstituted in FTOC. EGFP ^+^ cells purified on day 6 were analyzed for mRNA expression. Relative mRNA levels normalized to *GAPDH* levels are shown.
(171 KB PDF).

Figure S5 Antibody Array Analysis 293T cells transfected with HA-tagged IAN4 were lysed with buffer containing 0.5% Triton X-100. An antibody array membrane was incubated with the lysate and blotted with peroxidase-conjugated anti-HA antibody. An area of the array containing Bcl-2 family members is magnified in the box.
(310 KB PDF).

Figure S6 Subcellular Localization of IAN1, IAN4, and IAN5 (A) BW5147 cells expressing EGFP alone, EGFP-IAN1, EGFP-IAN4, or EGFP-IAN5 were stained with BODIPY-labeled BFA, MitoRed, or anti-CD44-PE.
(B) BW5147 cells expressing IAN1-HA, IAN4, IAN5, or EGFP were subjected to subcellular fractionation. Whole lysate (WL), nuclear (N), heavy membrane (HM) and cytoplasmic (C) fractions were analyzed by IB for IAN1-HA, IAN4, IAN5, EGFP, poly ADP-ribose polymerase (PARP), calnexin, cytochrome c oxidase 4 (COX4), Crk, or Bcl-2. PARP, calnexin, COX4, and Crk were enriched in the nuclei, endoplasmic reticulum, mitochondria, and cytoplasmic fractions, respectively, and used as markers for the fractionation.
(209 KB PDF).

Table S1 Oligonucleotide Microarray Analysis of *H-2 ^b^* AND-TCR–Transgenic Thymocytes and C57BL/6 Thymocytes
The fold difference of representative genes in two independent experiments is shown. Note that the expression of *IAN1* and *IAN4* is higher in AND-TCR–transgenic thymocytes than in C57BL/6 thymocytes. It was shown that *CCR7* is more highly expressed in mature SP thymocytes than in immature thymocytes [ 46], whereas *RAG1* and *RAG2* are more highly expressed in immature thymocytes than in mature thymocytes [ 47].
*CCR7,* CC chemokine receptor 7; *GAPDH,* glyceraldehyde 3-phosphate dehydrogenase; *RAG1* and *RAG2,* recombination activating genes 1 and 2; *Rps5,* ribosomal protein S5; *Tuba1,* tubulin alpha 1.
(14 KB PDF).

Table S2 Oligonucleotide Sequence and Predicted Secondary Structure of shRNA Double-stranded oligonucleotides encoding the sequences of the sense (19 nucleotides, underlined), the loop ( TTCAAGAGA, bold), the antisense (19 nucleotides, underlined), and the terminator ( TTTTTT) were synthesized to design shRNA, and were cloned downstream of a PolIII-dependent U6 promoter in the pSIREN-RetroQ vector (BD Clontech). The sequences of the synthesized oligonucleotides and the predicted secondary structure of the shRNA are listed.
(18 KB PDF).

### Accession Numbers

The Genbank ( http://www.ncbi.nih.gov/Genbank) accession numbers for the genes and gene products discussed in this paper are: mouse *IAN1*/ *gimap4* (NM_174990.3), mouse *IAN2*/ *gimap1* (NM_008376.3), mouse *IAN3*/ *gimap7* (NM_146167.3), mouse *IAN4*/ *gimap3* (AB164418.1), mouse *IAN5*/ *gimap5* (AB126961.1), mouse *IAN6*/ *gimap6* (NM_153175.3), mouse *IAN7*/ *gimap9* (BC096680.1), mouse *IAN9*/ *gimap8* (AB178029), rat *IAN1*/ *gimap4* (NM_173153), human *IAN1*/ *gimap4* (NM_018326), mouse *GAPDH* (M32599.1), mouse *bcl-2* (L31532.1), human *bcl-xL* (Z23115.1), human *bax* (NM_138761.2), human *bak* (NM_001188.2), human *bad* (NM_004322.2), human *bimEL* (AF032457.1), and human *IκBα*(AY033600.1).

## Abbreviations

DN: double negative
DP: double positive
EGFP: enhanced green fluorescent protein
FTOC: fetal thymus organ culture
GIMAP: GTPase of the immunity-associated protein
IAN: immune-associated nucleotide-binding protein
IB: immunoblotted
IP: immunoprecipitated
PCR: polymerase chain reaction
shRNA: short hairpin RNA
SP: single positive
TCR: T cell receptor
