# Supplementary material for: IAN Family Critically Regulates Survival and Development of T Lymphocytes
Source: PLoS Biol. 2006 Mar 7;4(4):e103. doi: 10.1371/journal.pbio.0040103 (PMC1393758; doi:10.1371/journal.pbio.0040103)
Supplement: Supplementary file 1 [file pbio.0040103.sg001.pdf]

Figure S1

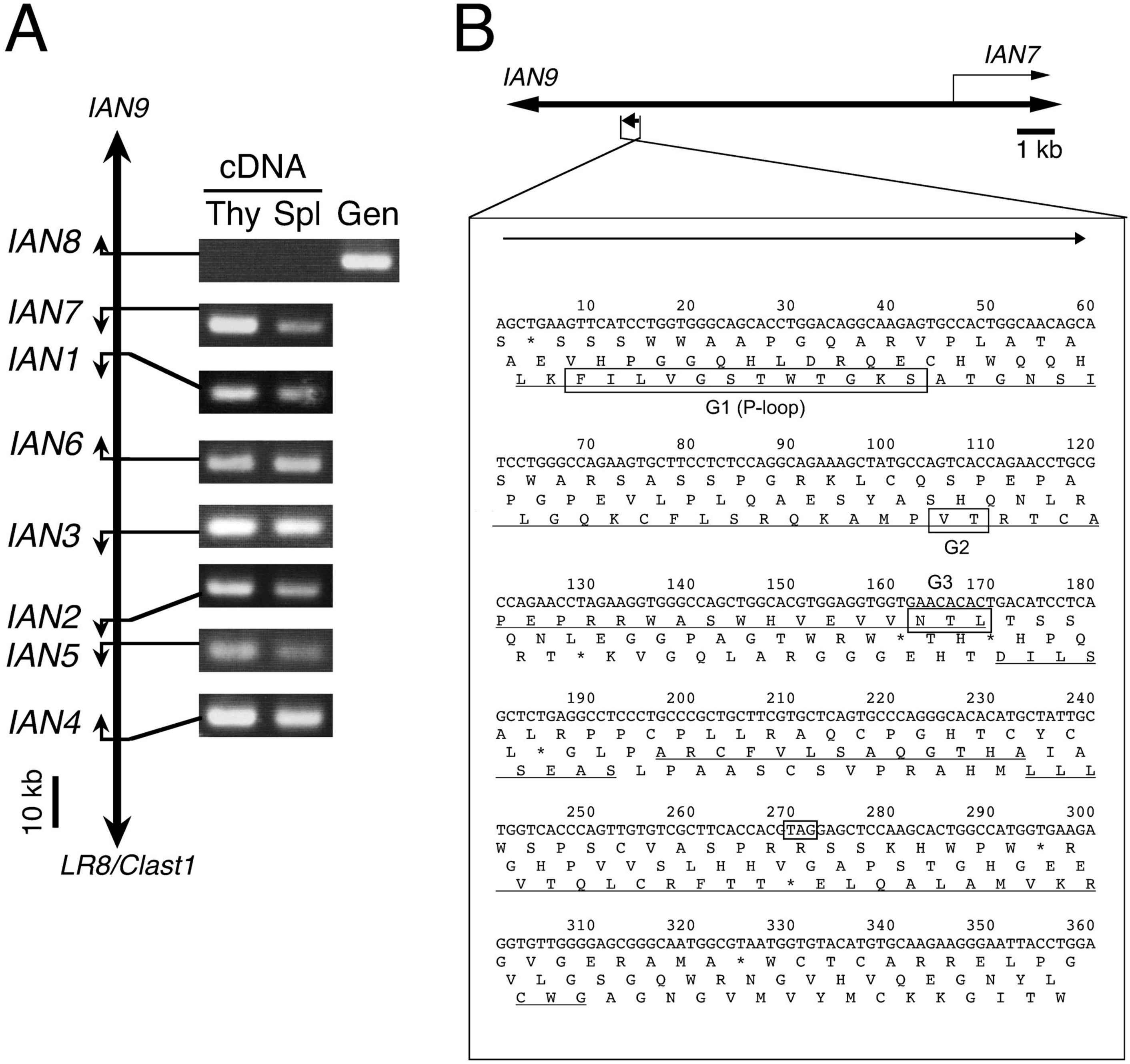

A
IAN9
cDNA
Thy
Spl
Gen
IAN8
IAN7
IAN1
IAN6
IAN3
IAN2
IAN5
IAN4
10 kb
LR8/Clast1
B
IAN9
IAN7
1 kb
10 20 30 40 50 60
AGCTGAAGTTCATCCTGGTGGGCAGCACCTGGACAGGCAAGAGTGCCACTGGCAACAGCA
S * S S S W W A A P G Q A R V P L A T A
A E V H P G G Q H L D R Q E C H W Q Q H
L K F I L V G S T W T G K S A T G N S I
G1 (P-loop)
70 80 90 100 110 120
TCCTGGGCCAGAAGTGCTTCCTCTCCAGGCAGAAAGCTATGCCAGTCACCAGAACCTGCG
S W A R S A S S P G R K L C Q S P E P A
P G P E V L P L Q A E S Y A S H Q N L R
L G Q K C F L S R Q K A M P V T R T C A
G2
G3
130 140 150 160 170 180
CCAGAACCTAGAAGGTGGGCCAGCTGGCACGTGGAGGTGGTGAACACACTGACATCCTCA
P E P R R W A S W H V E V V N T L T S S
Q N L E G G P A G T W R W * T H * H P Q
R T * K V G Q L A R G G G E H T D I L S
190 200 210 220 230 240
GCTCTGAGGCCTCCCTGCCCGCTGCTTCGTGCTCAGTGCCCAGGGCACACATGCTATTGC
A L R P P C P L L R A Q C P G H T C Y C
L * G L P A R C F V L S A Q G T H A I A
S E A S L P A A S C S V P R A H M L L L
250 260 270 280 290 300
TGGTCACCCAGTTGTGTCGCTTCACCACGTAGGAGCTCCAAGCACTGGCCATGGTGAAGA
W S P S C V A S P R R S S K H W P W * R
G H P V V S L H H V G A P S T G H G E E
V T Q L C R F T T * E L Q A L A M V K R
310 320 330 340 350 360
GGTGTTGGGGAGCGGGCAATGGCGTAATGGTGTACATGTGCAAGAAGGAATTACCTGGA
G V G E R A M A * W C T C A R R E L P G
V L G S G Q W R N G V H V Q E G N Y L
C W G A G N G V M V Y M C K K G I T W
