# Supplementary material for: IAN Family Critically Regulates Survival and Development of T Lymphocytes
Source: PLoS Biol. 2006 Mar 7;4(4):e103. doi: 10.1371/journal.pbio.0040103 (PMC1393758; doi:10.1371/journal.pbio.0040103)
Supplement: Supplementary file 2 [file pbio.0040103.sg002.pdf]

Figure S2

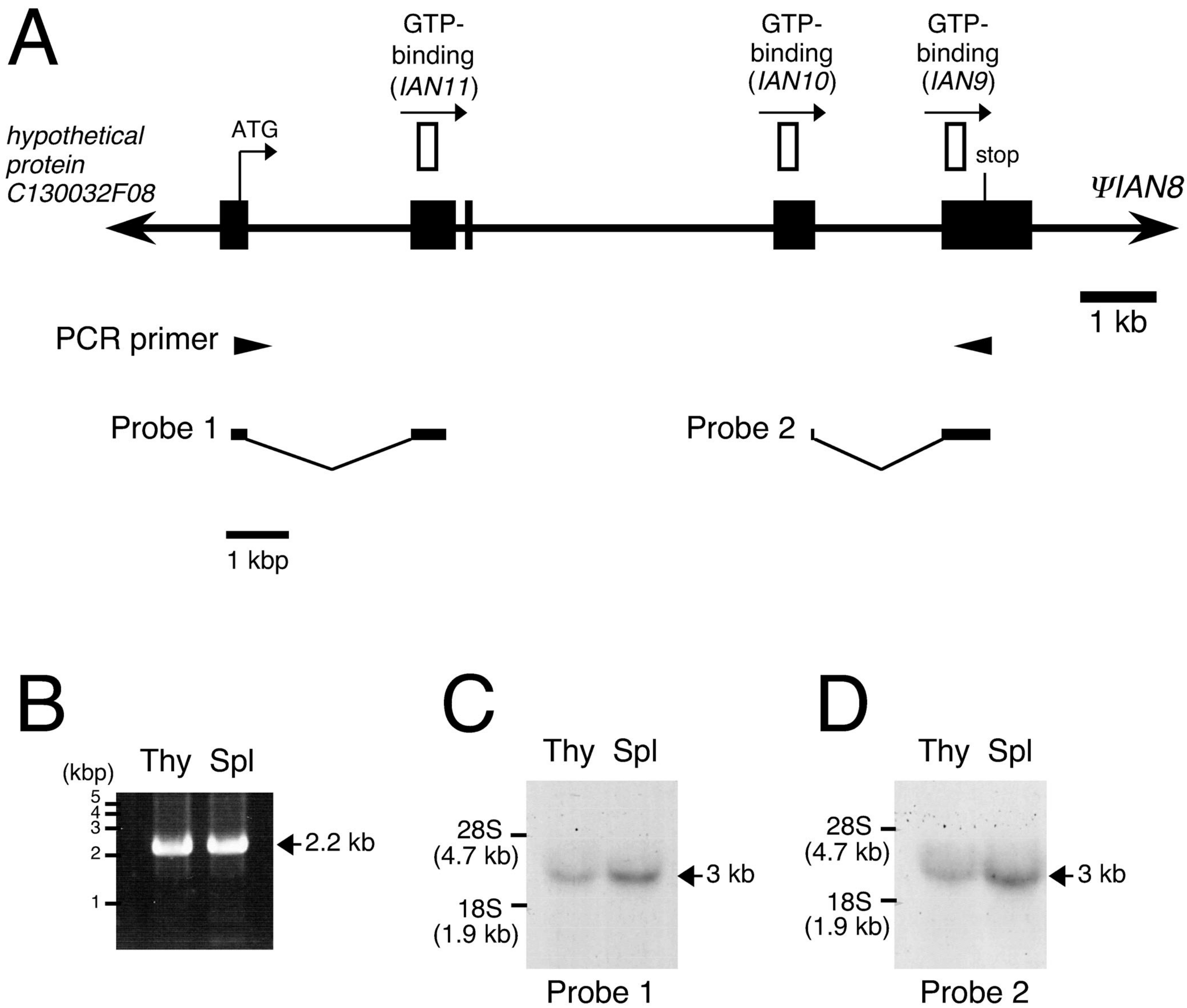
A
GTP-binding (IAN11)
GTP-binding (IAN10)
GTP-binding (IAN9)
hypothetical protein C130032F08
ATG
stop
ΨIAN8
1 kb
PCR primer
Probe 1
Probe 2
1 kbp
B
(kbp)
Thy
Spl
5
4
3
2
1
2.2 kb
C
Thy
Spl
28S (4.7 kb)
18S (1.9 kb)
3 kb
Probe 1
D
Thy
Spl
28S (4.7 kb)
18S (1.9 kb)
3 kb
Probe 2
