# Supplementary material for: IAN Family Critically Regulates Survival and Development of T Lymphocytes
Source: PLoS Biol. 2006 Mar 7;4(4):e103. doi: 10.1371/journal.pbio.0040103 (PMC1393758; doi:10.1371/journal.pbio.0040103)
Supplement: Supplementary file 3 [file pbio.0040103.sg003.pdf]

Figure S3

A

	% identical / amino acids							
	IAN1	IAN2	IAN3	IAN4	IAN5	IAN6	IAN7	IAN9
IAN1	100.0 / 328							
IAN2	37.9 / 235	100.0 / 300						
IAN3	37.2 / 288	39.2 / 240	100.0 / 293					
IAN4	36.5 / 233	40.1 / 287	41.4 / 220	100.0 / 301				
IAN5	38.3 / 240	47.1 / 210	42.9 / 217	83.8 / 291	100.0 / 308			
IAN6	38.8 / 206	43.7 / 197	39.5 / 195	50.3 / 183	50.6 / 180	100.0 / 305		
IAN7	39.3 / 270	38.4 / 203	53.1 / 290	37.6 / 229	37.6 / 229	41.4 / 203	100.0 / 291	
IAN9	32.7 / 223	41.1 / 192	42.3 / 189	39.9 / 203	39.9 / 203	41.1 / 180	39.8 / 186	100.0 / 688

B

```
                                           G1 (P-loop)
IAN4    1'     METLQNVVTGGKKGGCTSGSRPLRILLVGKSGCGKSATGNSLLRRPAFESRLRGQS
                         *    *   *  ******************* ****** *******
IAN5    1" MEHLQKSTYGTIVQGPEAHCVQESSCLRILLVGKSGCGKSATGNSILRRPAFQSRLRGQS

           G2                 G3
IAN4   57' VTRTSQAETGTWEGRSILVVDTPPIFESKAQNQDMDKDIGDCYLLCAPGHVLLLVTQLG
           ************************************************************
IAN5   61" VTRTSQAETGTWEGRSILVVDTPPIFESKAQNQDMDKDIGDCYLLCAPGHVLLLVTQLG

                                            G4
IAN4  117' RFTAEDVMAVRMVKEVFGVGVMRHMIVLFTRKEDLAEKSLEEFVTHTDNRSLRSLVQECG
           ****** **************************** ******************* ****
IAN5  121" RFTAEDAMAVRMVKEVFGVGVMRHMIVLFTRKEDLEEKSLEEFVTHTDNRSLRSLTQECG

            IAN motif        Coiled coil
IAN4  177' RRYCAFNNRASGEEQQGQLAELMALVRRLEQECEGSFHSNDLFLHAETLLREGYSVHQEA
           *********************************************** ************
IAN5  181" RRYCAFNNRASGEEQQGQLAELMALVRRLEQECEGSFHSNDLFLHAEALLREGYSVHQEA

                    Coiled coil                              Hydrophobic
IAN4  237' YRCYLAKVRQEVEKQRWELEEQEGSWVLKVLPIGKKLEVLHSDFCWYLVLAILIFFVFFF
           **************** *********  *     *     *   *  *    *     *
IAN5  241" YRCYLAKVRQEVEKQRRELEEQEGSWIAKMICTVKSCWSSHTAACALLIVLGLTLLTTFI

IAN4  297' LLFYV
            *
IAN5  301" NLCISRCK
```

C

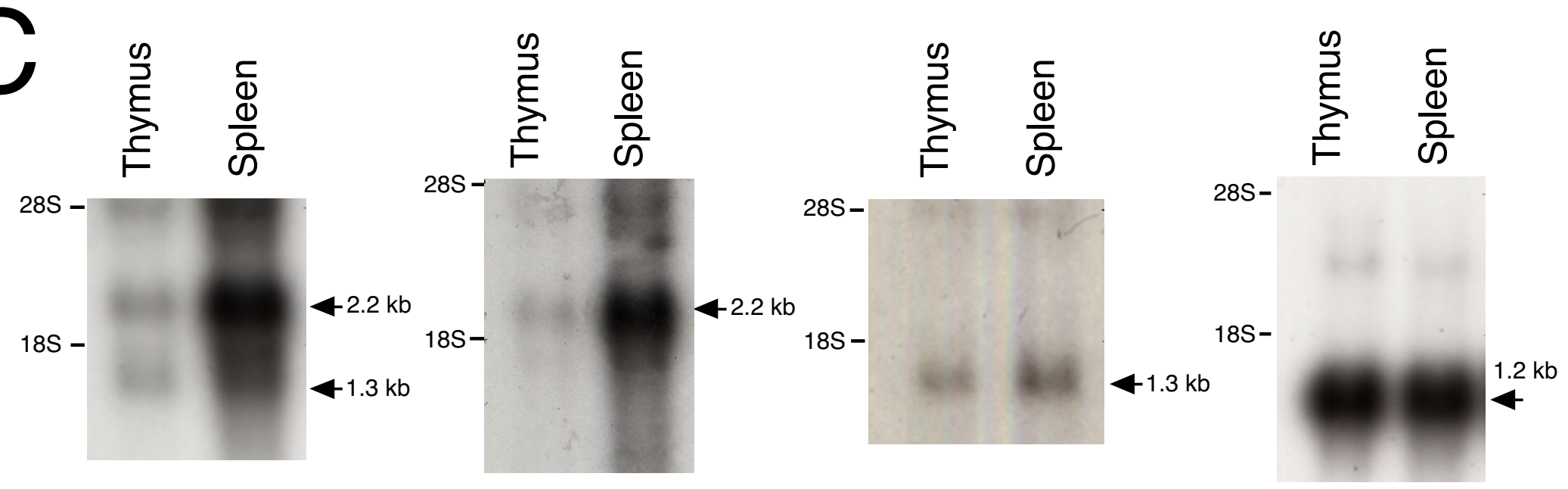

Probe: IAN4 ORF   IAN4-specific   IAN5-specific   GAPDH
