# Supplementary material for: IAN Family Critically Regulates Survival and Development of T Lymphocytes
Source: PLoS Biol. 2006 Mar 7;4(4):e103. doi: 10.1371/journal.pbio.0040103 (PMC1393758; doi:10.1371/journal.pbio.0040103)
Supplement: Supplementary file 4 [file pbio.0040103.sg004.pdf]

Figure S4

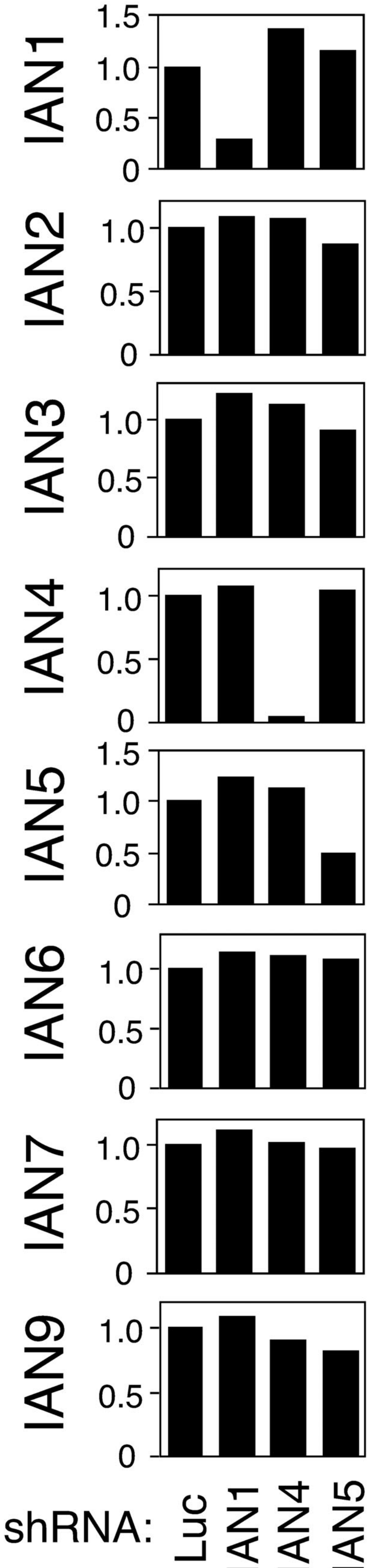
IAN1
1.5
1.0
0.5
0
IAN2
1.0
0.5
0
IAN3
1.0
0.5
0
IAN4
1.0
0.5
0
IAN5
1.5
1.0
0.5
0
IAN6
1.0
0.5
0
IAN7
1.0
0.5
0
IAN9
1.0
0.5
0
shRNA:
Luc
IAN1
IAN4
IAN5
