# Supplementary material for: IAN Family Critically Regulates Survival and Development of T Lymphocytes
Source: PLoS Biol. 2006 Mar 7;4(4):e103. doi: 10.1371/journal.pbio.0040103 (PMC1393758; doi:10.1371/journal.pbio.0040103)
Supplement: Supplementary file 5 [file pbio.0040103.sg005.pdf]

Figure S5

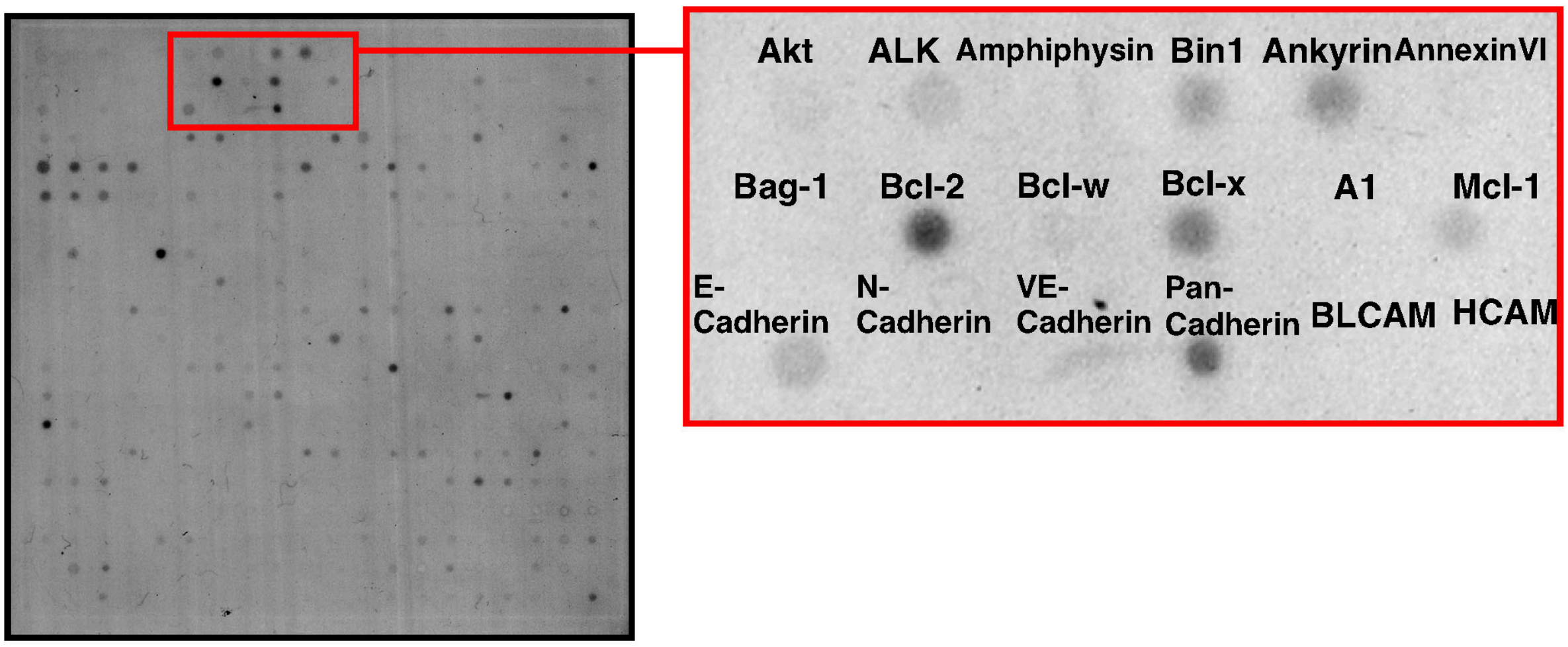
Akt
ALK
Amphiphysin
Bin1
Ankyrin
AnnexinVI
Bag-1
Bcl-2
Bcl-w
Bcl-x
A1
Mcl-1
E-Cadherin
N-Cadherin
VE-Cadherin
Pan-Cadherin
BLCAM
HCAM
