# Supplementary material for: IAN Family Critically Regulates Survival and Development of T Lymphocytes
Source: PLoS Biol. 2006 Mar 7;4(4):e103. doi: 10.1371/journal.pbio.0040103 (PMC1393758; doi:10.1371/journal.pbio.0040103)
Supplement: Supplementary file 6 [file pbio.0040103.sg006.pdf]

Figure S6

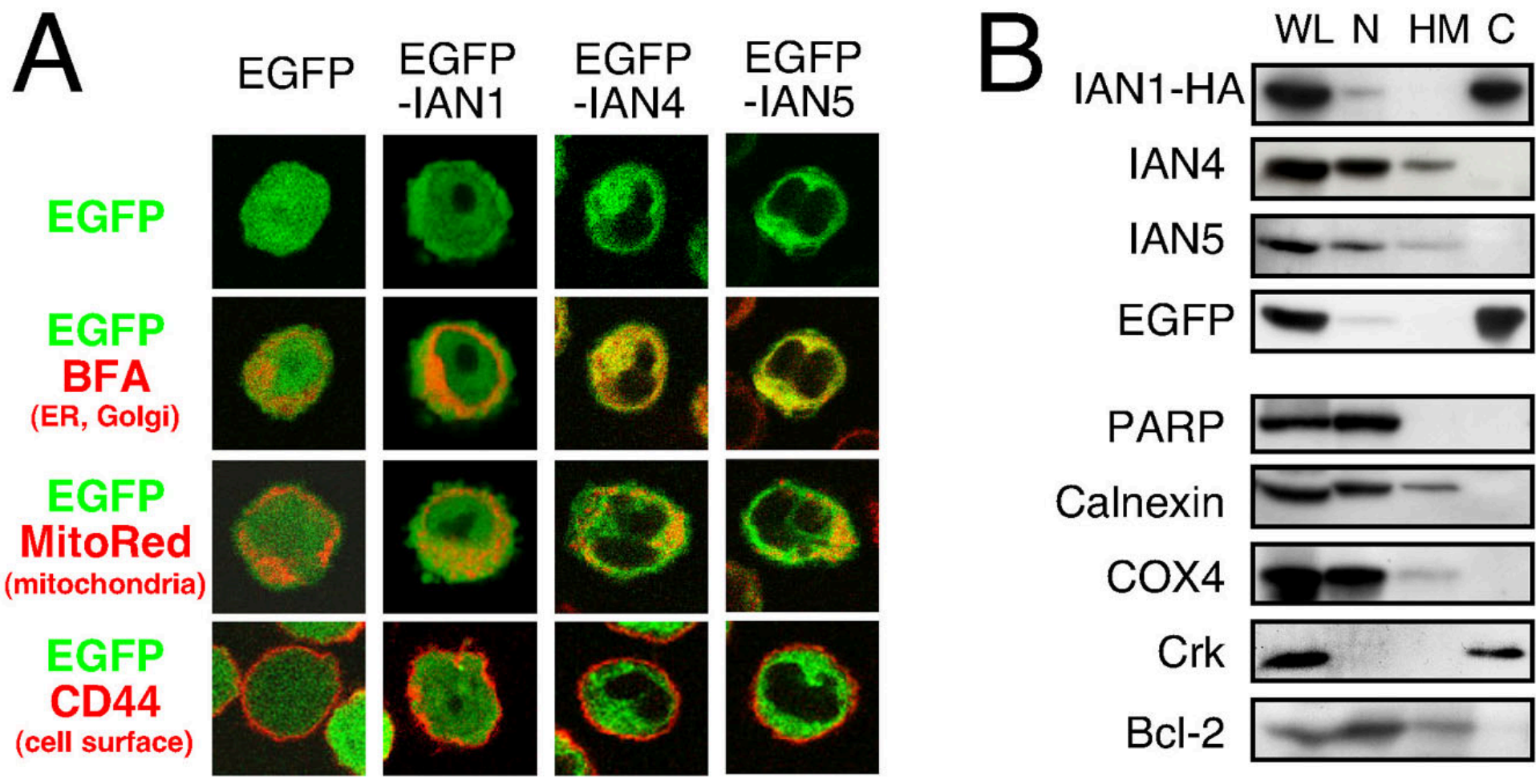
A
EGFP
EGFP -IAN1
EGFP -IAN4
EGFP -IAN5
EGFP
EGFP
BFA
(ER, Golgi)
EGFP
MitoRed
(mitochondria)
EGFP
CD44
(cell surface)
B
WL N HM C
IAN1-HA
IAN4
IAN5
EGFP
PARP
Calnexin
COX4
Crk
Bcl-2
