# Supplementary material for: IAN Family Critically Regulates Survival and Development of T Lymphocytes
Source: PLoS Biol. 2006 Mar 7;4(4):e103. doi: 10.1371/journal.pbio.0040103 (PMC1393758; doi:10.1371/journal.pbio.0040103)
Supplement: Supplementary file 7 [file pbio.0040103.st001.pdf]

## Table S1

| Accession No. | Gene | Fold increase (AND / B6) | | |
|---|---|---|---|---|
| | | Expt. 1 | Expt. 2 | Average |
| AA795946 | *IAN1* | 4.3 | 4.7 | 4.5 |
| AA958556 | *IAN4* | 3.3 | 2.7 | 3.0 |
| L31580 | *CCR7* | 3.6 | 4.2 | 3.9 |
| M32599 | *GAPDH* | 0.9 | 1.0 | 1.0 |
| M28729 | *Tuba1* | 0.9 | 1.0 | 1.0 |
| AV140484 | *Rps5* | 0.9 | 1.0 | 1.0 |
| M29475 | *RAG1* | 0.2 | 0.2 | 0.2 |
| M64796 | *RAG2* | 0.2 | 0.1 | 0.2 |
