# Supplementary material for: IAN Family Critically Regulates Survival and Development of T Lymphocytes
Source: PLoS Biol. 2006 Mar 7;4(4):e103. doi: 10.1371/journal.pbio.0040103 (PMC1393758; doi:10.1371/journal.pbio.0040103)
Supplement: Supplementary file 8 [file pbio.0040103.st002.pdf]

Table S2

Gene	Accession no.	Sequence of double-stranded oligonucleotides	Predicted secondary structure of shRNA
Firefly *luciferase*	M15077	5'-GATCCGTGCGTTGCTAGTACCAAC**TTCAAGAGA**GTTGGTACTAGCAACGCACTTTTTTG -3'<br>3'- GCACGCAACGATCATGGTTG**AAGTTCTCT**CAACCATGATCGTTGCGTGAAAAAACTTAA-5'	5'- GUGCGUUGCUAGUACCAAC **U** **UCA** **A**<br>3'-UUCACGCAACGAUCAUGGUUG **A** **GAG**
Mouse *IAN1*	NM_174990	5'-GATCCGTGCCGAGTGTGTGATCCA**TTCAAGAGA**TGGATCACACACTCGGCACTTTTTTG -3'<br>3'- GCACGGCTCACACACTAGGT**AAGTTCTCT**ACCTAGTGTGTGAGCCGTGAAAAAACTTAA-5'	5'- GUGCCGAGUGUGUGAUCCA **U** **UCA** **A**<br>3'-UUCACGGCUCACACACUAGGU **A** **GAG**
Mouse *IAN4*	AB164418	5'-GATCCGTTGGAGGTGTTGCATTCA**TTCAAGAGA**TGAATGCAACACCTCCAACTTTTTTG -3'<br>3'- GCAACCTCCACAACGTAAGT**AAGTTCTCT**ACTTACGTTGTGGAGGTTGAAAAAACTTAA-5'	5'- GUUGGAGGUGUUGCAUUCA **U** **UCA** **A**<br>3'-UUCAACCUCCACAACGUAAGU **A** **GAG**
Mouse *IAN5*	AB126961	5'-GATCCGTCCTGCTGGAGCTCCCAC**TTCAAGAGA**GTGGGAGCTCCAGCAGGACTTTTTTG -3'<br>3'- GCAGGACGACCTCGAGGGTG**AAGTTCTCT**CACCCTCGAGGTCGTCCTGAAAAAACTTAA-5'	5'- GUCCUGCUGGAGCUCCCAC **U** **UCA** **A**<br>3'-UUCAGGACGACCUCGAGGGUG **A** **GAG**
